# AC‐1001 H3 CDR peptide induces apoptosis and signs of autophagy *in vitro* and exhibits antimetastatic activity in a syngeneic melanoma model

**DOI:** 10.1002/2211-5463.12080

**Published:** 2016-07-15

**Authors:** Aline N. Rabaça, Denise C. Arruda, Carlos R. Figueiredo, Mariana H. Massaoka, Camyla F. Farias, Dayane B. Tada, Vera C. Maia, Pedro I. Silva Junior, Natalia Girola, Fernando Real, Renato A. Mortara, Luciano Polonelli, Luiz R. Travassos

**Affiliations:** ^1^ Unidade de Oncologia Experimental (UNONEX)Universidade Federal de São Paulo (UNIFESP)Brazil; ^2^ Núcleo Integrado de BiotecnologiaUniversidade de Mogi das CruzesBrazil; ^3^ Departamento de Ciência e TecnologiaUniversidade Federal de São Paulo (UNIFESP)São José dos CamposBrazil; ^4^ Recepta BiopharmaSão PauloBrazil; ^5^ Laboratório Especial de Toxinologia AplicadaInstituto ButantanSão PauloBrazil; ^6^ Departamento de ParasitologiaUniversidade Federal de São Paulo (UNIFESP)Brazil; ^7^ Microbiology and Virology UnitDepartment of BiomedicalBiotechnological and Translational SciencesUniversitá degli Studi di ParmaItaly

**Keywords:** apoptosis, autophagy, immunoglobulin CDR, melanoma, peptides

## Abstract

Antibody‐derived peptides modulate functions of the immune system and are a source of anti‐infective and antitumor substances. Recent studies have shown that they comprise amino acid sequences of immunoglobulin complementarity‐determining regions, but also fragments of constant regions. V_H_ CDR3 of murine mAb AC‐1001 displays antimetastatic activities using B16F10‐Nex2 murine melanoma cells in a syngeneic model. The peptide was cytotoxic *in vitro* in murine and human melanoma cells inducing reactive oxygen species (ROS) and apoptosis by the intrinsic pathway. Signs of autophagy were also suggested by the increased expression of LC3/LC3II and Beclin 1 and by ultrastructural evidence. AC‐1001 H3 bound to both G‐ and F‐actin and inhibited tumor cell migration. These results are important evidence of the antitumor activity of Ig CDR‐derived peptides.

**Abbreviations**
CDR: complementary determining regions
DHE: dihydroethidium
Ig‐V_H_: heavy chain variable region on immunoglobulin
MTT: 3‐[4,5‐dimethylthiazol‐2‐yl]‐2,5‐diphenyltetrazolium bromide
pNA: para‐nitroaniline
ROS: reactive oxygen species
TEM: transmission electron microscopy
TMRE: tetramethylrhodamine ethyl ester
TUNEL: terminal deoxynucleotidyl transferase dUTP nick end‐labeling

Melanoma is the most aggressive skin cancer 1 responsible for 80% of lethal cases 2, 3. The malignant tumor arises from transformed melanocytes, which originate from the neural‐crest and accumulate genomic mutations leading to uncontrolled proliferation, invasion, and metastasis 4, 5. Melanoma stem cells can also be isolated representing a population highly enriched in the neural‐crest growth factor receptor (CD271) 6. Conventional anticancer therapy has not been effective in advanced melanoma 4, 7. Immune checkpoint monoclonal antibodies are encouraging therapeutic agents being tested against the metastatic tumor 8.

Anticancer peptides from natural sources are emerging as potential therapeutic agents, mainly because of their small size, low immunogenicity, better tissue penetration, and easy production, with the possibility of low‐cost structural modifications. They can display a wide range of effects, such as necrotic, apoptotic, function blocking, anti‐angiogenic, and immunostimulatory activities 9.

In a collaborative study, Polonelli *et al*. 10 showed that immunoglubulins may represent an unlimited source of bioactive peptides, particularly those corresponding to the complementarity‐determining regions (CDRs). Some of these CDR‐derived peptides have been shown to display differential antimicrobial, antiviral, immunomodulatory, and antitumor activities, independently of the specificity of the native antibody 10, 11. In particular, the peptides based on the CDR 3 of the heavy chain (H3), could act as micro‐antibodies and reproduce some properties of the original immunoglobulin 12, 13.

Polonelli *et al*. 10 showed that the synthetic peptide corresponding to the human mAb HuA CDR L1, but not the mAb itself specific for difucosyl human blood group A, caused apoptosis *in vitro* on melanoma cells and exerted antimetastatic effects *in vivo* 10. Human (HuA) and mouse (AC‐1001) monoclonal antibodies to blood group A recognize the same epitope although they have completely different sequences 14. A recent study established that the noncandidacidal synthetic peptide with the sequence of the CDR H3 of mAb AC‐1001 exhibited antifungal effect against systemic candidiasis based on an immunomodulatory effect 15.

Based on the cross‐cytotoxicity of CDR peptides using different cell targets, we aimed, in this study, at investigating the antitumor activity of AC‐1001 H3 *in vitro* and *in vivo*. The peptide induced apoptosis and signs of autophagy *in vitro*, using B16F10‐Nex2 melanoma cells, targeting β‐actin. Furthermore, the peptide inhibited tumor cell metastasis *in vivo*, in a syngeneic mouse model, standing as a potential anticancer drug.

## Materials and methods

### Cell lines

The human cell lines A2058 (melanoma) and the murine cell line B16F10 (melanoma) were provided by the Ludwig Institute for Cancer Research, São Paulo, Brazil. The subline B16F10‐Nex2 was established at the Experimental Oncology Unit, Federal University of São Paulo, as described previously 16 and is deposited at Banco de Células do Rio de Janeiro, BCRJ No. 0342. The human umbilical vein endothelial cell (HUVEC) was provided by Prof. Helena Nader, Federal University of São Paulo and the human fibroblast cell (T75) was provided by Prof. Oswaldo Keith Okamoto, University of São Paulo. Cell lines were maintained in RPMI 1640 (Gibco, Grand Island, NY, USA) or DMEM (Gibco) supplemented with 10 mm HEPES (Sigma‐Aldrich, St Louis, MO, USA), 24 mm sodium bicarbonate, 40 mg·L^−1^ gentamicin (Hipolabor, Sabará, MG, Brazil), pH 7.2, and 10% fetal bovine serum (Gibco) at 37 °C in a humidified atmosphere containing 5% CO_2_.

### Peptides

Peptide AC‐1001 H3 (GQYGNLWFAY‐NH_2_) and the scrambled peptide (YAFGWNQLYG‐NH_2_) were synthesized by Peptide 2.0 (Chantilly, VA, USA) and diluted in 2% DMSO, 20% Milli‐Q‐distilled water, and RPMI 1640 medium in this order for stable solubility.

### Cell viability assay

Cells were cultivated (10^4^/well) in 96‐well plates and incubated with AC‐1001 H3 or with scrambled peptide at different concentrations: 0, 0.08, 0.175, and 0.35 mm at 37 °C in a humidified atmosphere containing 5% CO_2_. After 18 h incubation, MTT (3‐[4,5‐dimethylthiazol‐2‐yl]‐2,5‐diphenyltetrazolium bromide) (Sigma‐Aldrich) solution (5 mg·mL^−1^) was added to each well and incubated at 37 °C. After 3 h, 10% SDS was directly added to dissolve the formazan crystals and, on the following day, absorbance was measured using an automated spectrophotometric plate reader (SpectraMax‐M2, Software Pro 5.4; Molecular Devices, Sunnyvale, CA, USA) at 570 nm. Cell viability is expressed as percent values in comparison with untreated cells.

### Superoxide anions

Superoxide anions production was determined by dihydroethidium (DHE) assay according to the manufacturer's instructions (Invitrogen, Carlsbad, CA, USA). Briefly, B16F10‐Nex2 cells (10^4^/well) were cultivated in 48‐well plates, treated with 0.35 mm AC‐1001 H3 or medium (control) during 18 h at 37 °C and then incubated with 5 μm DHE for 30 min at room temperature. Cells were treated with 5 mm H_2_O_2_ at 37 °C for 20 min as a positive control. The cells were immediately analyzed by fluorescence microscopy in an Olympus BX‐51 microscope using a 40× objective. DHE oxidation was observed by the red fluorescence. Images were processed with imagej.

### Chromatin condensation

The chromatin condensation analysis was determined using Hoechst 33342 (Invitrogen), which intercalates within DNA emitting blue fluorescence. B16F10‐Nex2 cells (10^4^/well) were cultivated in 48‐well plates and treated with 0.35 mm AC‐1001 H3 or medium (control) during 18 h at 37 °C. They were then fixed with 2% formaldehyde for 30 min and stained with 2 μm Hoechst 33342 for 10 min at room temperature. The cells were analyzed by fluorescence microscopy in an Olympus BX‐51 microscope using a 20× objective. Apoptotic cells were detected by chromatin condensation. Images were processed with imagej.

### TUNEL assay

The TUNEL (terminal deoxynucleotidyl transferase dUTP nick end‐labeling) assay, detects DNA fragmentation by labeling the terminal end of nucleic acids. It was used according to the manufacturer's protocol (‘*In Situ* Cell Death Detection kit, AP’; Roche Molecular Biochemicals, Mannheim, Germany). Briefly, B16F10‐Nex2 cells (10^4^/well) were cultivated in 48‐well plates and treated with 0.35 mm AC‐1001 H3 or medium (control) for 18 h at 37 °C. Treatment with 2 μg·mL^−1^ Actinomycin D (Sigma‐Aldrich) for 2 h was used as a positive control. Cells were fixed with 2% formaldehyde for 30 min and were permeabilized with 0.1% Triton X‐100 for 30 min at room temperature. After washing, the cells were incubated with 50 μL of TUNEL reaction mixture for 1 h, at 37 °C and protected from light. These cells were also stained with 10 μg·mL^−1^ DAPI (Invitrogen) for 10 min. The cells were analyzed by fluorescence microscopy in an Olympus BX‐51 microscope using a 20× objective. Images were processed with imagej.

### Caspase activation

Activation of caspases 3, 8, and 9 was determined by the Apotarget Caspase Colorimetric Protease Assay Sampler Kit (Invitrogen) according to the manufacturer's instructions. In summary, B16F10‐Nex2 cells (3 × 10^5^/well) were cultivated in 6‐well plates and treated with 0.35 mm AC‐1001 H3 or medium (control) during 12 h at 37 °C. After washing, they were harvested, pelleted, and resuspended in 50 μL of chilled cell lysis buffer and incubated in ice for 10 min. The lysate was centrifuged at 10 000 ***g*** for 1 min and the supernatant was transferred to a fresh tube. Bradford method was used to determine the protein concentration and the extract was diluted to 3 mg·mL^−1^. An equal volume (50 μL) of 2× Reaction Buffer with 10 mm DTT was added to each sample. The samples were incubated with 200 μm of the substrates, DEVD‐pNA (caspase‐3), IETD‐pNA (caspase‐8), and LEHD‐pNA (caspase‐9), at 37 °C for 2 h in a 96‐well plate. The absorption of light by free para‐nitroaniline (pNA) as a result of the synthetic substrates‐pNA cleavage by caspases was quantified using a microplate reader (SpectraMax‐M2, Software Pro 5.4; Molecular Devices) at 405 nm.

### Morphological alterations – transmission electron microscopy

B16F10‐Nex2 cells (10^4^/well) were cultivated in 24‐well plates and treated with 0.35 mm AC‐1001 H3 overnight at 37 °C. They were fixed in a solution of 2.5% glutaraldehyde and 2% formaldehyde in 0.1 m sodium cacodylate buffer, pH 7.2, at room temperature for 3 h. After washing in the same buffer for 10 min, they were fixed with 1% osmium tetroxide in 0.1 m cacodylate at pH7.2 for 30 min, and washed with water for 10 min at room temperature. Subsequently, cells were treated with aqueous 0.4% uranyl acetate for 30 min and washed again for 10 min. Cells were then dehydrated in graded ethanol (70%, 90%, and 100%), treated quickly with propylene oxide, and embedded in SPURR. Ultrathin sections were collected on grids and stained in alcoholic 1% uranyl acetate and in lead citrate prior to examination in a Jeol 100 CX electron microscope (Tokyo, Japan) to investigate the morphological alterations induced by AC‐1001 H3 treatment.

### Phosphatidylserine translocation

The ‘Annexin V‐FITC Apoptosis Detection Kit’ (Sigma‐Aldrich), that measures the binding of annexin V‐FITC to translocated phosphatidylserine in the membrane of apoptotic cells and the binding of propidium iodide to DNA when the cell membrane has been compromised (necrotic cells), was used to identify apoptotic and necrotic cells by flow cytometry. B16F10‐Nex2 cells (2 × 10^5^/well) were cultivated in 6‐well plates and treated with 0.35 mm AC‐1001 H3 or RPMI with 2% DMSO during 12 h at 37 °C. After washing, they were harvested with a cell scraper, pelleted and washed again twice. The samples were then resuspended in binding buffer (10 mm HEPES/NaOH, pH 7.5, 140 mm NaCl, and 2.5 mm CaCl2) in the presence of propidium iodide (0.5 μg·mL^−1^) and annexin V (2 μg·mL^−1^) for 10 min at room temperature in the dark and immediately analyzed by flow cytometry (FACSCanto II; BD Bioscience, Franklin Lakes, NJ, USA; using facsdiva software; BD Bioscience and flowjo software; TreeStar Inc., Ashland, OR, USA).

### Mitochondrial membrane‐potential disruption

B16F10‐Nex2 cells (2 × 10^5^/well) were cultivated in 6‐well plates and treated with 0.35 mm AC‐1001 H3, medium (control) or the scrambled peptide overnight at 37 °C. Cells were gently washed, harvested with PBS‐EDTA, pelleted, and stained with 20 nm tetramethylrhodamine ethyl ester (TMRE; Molecular Probes, Eugene, OR, USA) for 10 min at 37 °C and protected from light. TMRE is a cationic lipophilic dye that accumulates in active mitochondria (negatively charged) and is unable to be sequestered in depolarized or inactive mitochondria. Fluorescence was measured by FACSCanto II (BD Bioscience), using facsdiva software (BD Bioscience) and analyzed using flowjo software (TreeStar Inc.).

### LC3 and Beclin 1 labeling

Autophagy phenotypic traits were analyzed through confocal microscopy, using antibodies against LC3 and Beclin proteins, essential for the formation of autophagosomes. B16F10‐Nex2 cells (10^4^) were cultivated in round glass coverslips and treated with 0.35 mm AC‐1001 H3 or medium with 2% DMSO (control) overnight at 37 °C. They were fixed with 2% formaldehyde for 30 min and permeabilized with Triton X‐100 0.1% for 30 min at room temperature. After blocking with immunofluorescence buffer (150 mm NaCl, 50 mm Tris, 0.1% Tween, and 0.25% BSA) for 1 h, at 37 °C, they were incubated with 1 : 500 anti‐LC3 or anti‐Beclin (both from Sigma‐Aldrich) for 3 h, following incubation with 1 : 1000 anti‐rabbit‐AlexaFluor 488 (Invitrogen, Eugene, OR, USA) for 1.5 h. These cells were also stained with 10 μg·mL^−1^ DAPI (Invitrogen) for 10 min. The coverslips were mounted on slides with 4 μL of Vectashield (Sigma) and observed in a Confocal Leica SP5 microscope, with a 63 × 1.4 oil objective; the Z series was obtained according with sampling criteria built in the software. Images were processed using imagej.

### Western blotting

B16F10‐Nex2 cells were treated with AC‐1001 H3 (0.35 mm) or medium (control) overnight, washed with PBS and lysed for 15 min at 4 °C with ice cold RIPA buffer (50 mm Tris‐Cl, pH 7.5, 150 mm NaCl, 1% Nonidet P‐40, 0.5% sodium deoxycholate, and 0.1% SDS) supplemented with protease and phosphatase inhibitors (Sigma‐Aldrich). After sonication, samples were centrifuged and the supernatants were collected. The protein concentration of lysates was measured using Bradford method. Proteins from each cell lysate were separated by SDS gel electrophoresis and transferred to a nitrocellulose membrane (Millipore, Billerica, MA, USA), which was blocked in TPBS (PBS with 0.05% Tween‐20) with 5% skim milk. After washing, the membranes were incubated overnight at 4 °C with 1 : 1000 of primary antibody anti‐LC3 (Sigma‐Aldrich) and anti‐GAPDH (Millipore, Temecula, CA, USA) was used as loading control. Then, they were washed again and incubated for 1 h at room temperature with the secondary antibody anti‐rabbit IgG‐HRP and anti‐mouse IgG‐HRP, respectively (both from Cell Signaling Technology, Beverly, MA, USA). Protein band were detected using the UVItec Alliance Gel Documentation System (UVItec, Cambridge, UK). Band area intensity was quantified using imagej.

For an expression kinetics of Beclin 1 and caspase 3, B16F10‐Nex2, and A2058 cells (5 × 10^4^) were treated with 0.35 mm of AC1001 peptide for 4, 8 and 16 h. After treatment, proteins in cell lysates were analyzed by western blotting as described elsewhere 17. The following antibodies were used: anti‐α‐tubulin (Sigma Aldrich), anti‐Beclin 1, anti‐caspase‐3 or cleaved caspase‐3 purchased from Cell Signaling Technology. α‐tubulin was used as loading control. Secondary antibodies conjugated with IgG horseradish peroxidase were purchased from Cell Signaling Technology.

### LysoTracker Red labeling

B16F10‐Nex2 cells were treated with 0.35 mm AC‐1001 H3 or medium (control), simultaneously labeled with 5 μm LysoTracker Red (Invitrogen) and immediately placed on a Confocal Leica SP5 microscope, with a 63 × 1.4 oil objective. Images were taken every 5 min during 22 h, processed using imagej and analysis were done using imaris (Bitplane AG, Zurich, Switzerland).

### Mass spectrometry

The molecular target of the peptide was identified by mass spectrometry. B16F10‐Nex2 cells (5 × 10^8^) were incubated with a lysis buffer (1% Triton X‐100, 10 mm Tris‐HCl, 5 mm EDTA, 50 mm NaCl, 200 μm oxidized glutathione, pH 7.6) and a mixture of protease inhibitors (Pierce, Rockford, IL, USA) and it was incubated overnight at 4 °C with biotinylated AC‐1001 H3. The lysate was passed through a column of streptavidin‐agarose (Pierce) and eluted with 2% urea. The eluate was separated in 10% SDS/PAGE, and the band detected with colloidal Coomassie Blue was sliced and digested with trypsin (Promega, Fitchburg, WI, USA) as described elsewhere 18. An aliquot of the digested protein (4.5 μL) was injected in the analytic column C18 1.7‐μm BEH 130 (100 μm × 100 mm) RP‐UPLC (nanoAcquity UPLC; Waters, Milford, MA, USA) coupled to a Q‐TOF Ultima API mass spectrometer (Micro Mass; Waters) at a flow rate of 600 nL·min^−1^. A trapping column Symmetry C18 (180 μm × 20 mm) was used for sample desalting at a flow rate of 20 μL·min^−1^ over 1 min. The gradient was 0–50% acetonitrile in 0.1% formic acid over 45 min. The instrument was operated in MS positive mode, data continuum acquisition from *m*/*z* 100–2000 Da at a scan rate of 1 s, and an interscan delay of 0.1 s. Database searches for peptide identification from LC MS/MS experiments were done with mascot distiller v.2.3.2.0, 2009 (Matrix Science, Boston, MA, USA) using carbamidomethyl‐cys as a fixed modification (monoisotopic mass 57.0215 Da), methionine oxidation as variable modification (monoisotopic mass 15.9949), and 0.1 Da MS and MS/MS fragment size tolerance.

### ELISA

The ELISA was performed to determine the binding of AC‐1001 H3 peptide to β‐actin. A 96‐well opaque plate (Nunc, Roskilde, Denmark) was coated with AC‐1001 H3 or scrambled peptide (500 μg·mL^−1^) diluted in carbonate‐bicarbonate buffer, pH 9.2. In the following day, the plate was washed 5× with 0.1% PBS‐Tween (T‐PBS) and 5 m NaCl and blocked at 4 °C with 1% BSA. After 2 h, the plate was washed again and G‐actin or F‐actin (Cytoskeleton, Denver, CO, USA) was added at 1 : 500 and incubated for 4 h. Anti‐actin antibody (Novus Biologicals, Littleton, CO, USA), at 1 : 1000, was then added for 2 h. The plate was extensively washed and the secondary anti‐mouse IgG‐peroxidase (Sigma‐Aldrich) was added (1 : 1000) for 1 h. After washing with PBS, the reaction was evaluated by chemoluminescence using ECL (1 : 500; Millipore) in a luminometer (SpectraMax) at 470 nm.

### Synthesis of PLGA nanoparticles

The PLGA Nanoparticles (NPs) containing fluorescent probe (RhodamineB; Sigma‐Aldrich R6626) were synthesized by oil in water emulsification and solvent evaporation method. Initially, 500 μL of Rhodamine B aqueous solution (0.5 mm) were added to 20 mL of aqueous solution of PVA 1% (w/v). Following, 4 mL of PLGA amino terminated solution in dimethylformamide [2.5% (w/v)] was poured into the PVA/rhodamine solution under stirring. The emulsion was stirred at room temperature for 17 h. Then, in order to eliminate microparticles, the suspension was centrifuged (200 ***g***, 4 °C, 15 min) and the precipitate was discarded. Finally, NPs were collected by centrifugation (44 100 ***g***, 15 min, 4 °C). After washing four times with distilled water, the NPs were suspended in 27 mL of phosphate buffer saline (PBS). 13.5 mL of NPs suspension was stored at − 4 °C and these NPs were used as blank control. The other 13.5 mL of NPs suspension were used in the coupling reaction with AC‐1001 H3 peptide (GQYGNLWFAY).

### Coupling AC‐1001 H3 to PLGA NPS

Prior to the addition of peptide, PLGA NPs surface reacted with 1‐ethyl‐3‐[3‐dimethylaminopropyl] carbodiimide hydrochloride (EDC) (Sigma) and N‐hydroxysuccinimide (NHS) (Sigma). Firstly, NPs were centrifuged and resuspended in 13.5 mL of borate buffer solution (pH 7.3),and then 30 μL of aqueous solution of EDC (1 mm) and NHS (1 mm) were added to the NPs suspension. After 1 h under stirring, NPs were centrifuged (44 100 ***g***, 15 min, 4 °C) and resuspended in autoclaved water. Then, 300 μL of peptide solution (0.75 mm) were added and the mixture was stirred for 2 h. Finally, NPs‐peptide was centrifuged and resuspended in 13.5 mL of sterilized PBS.

### Peptide‐NPs‐Rhodamine confocal microscopy

B16F10‐Nex2 cells (10^4^) were cultivated in round glass coverslips and treated with AC‐1001‐NPs‐Rhodamine solution for 3 h protected from light at 37 °C. They were then fixed with 4% formaldehyde for 30 min and permeabilized with Triton X‐100 0.1% for 30 min at room temperature. These cells were also stained with 1 : 200 Phalloidin‐FITC (Molecular Probes) during 1 h at 37 °C in the dark and 1 mg·mL^−1^ DAPI (Invitrogen) for 10 min. The coverslips were mounted on slides with 4 μL of Vectashield (Sigma) and observed in a Confocal Leica SP5 microscope, with a 63 × 1.4 oil objective; DAPI was examined at 350 nm excitation and 470 nm emission, Phalloidin‐FITC at 490/520 nm and Peptide‐Rhodamine at 580/604 nm, respectively. The Z series was obtained according with sampling criteria built in the software. Images were processed using imagej.

### Animals and *in vivo* metastatic model

Six‐ to eight‐week‐old male C57BL/6 mice were obtained from the Center for Development of Experimental Models (CEDEME, Federal University of São Paulo (UNIFESP), and kept in isolators, with autoclaved water and food. Animal experiments were carried out in accordance with the UNIFESP Ethics Committee for Animal Experimentation (CEP No. 1807‐11). Syngeneic B16F10‐Nex2 cells (5 × 10^5^) cells were injected (in 0.1 mL RPMI without SFB) intravenously in the tail veins of mice (*n* = 5/group) and in the following day the intraperitoneal treatment (10 consecutive days) with AC‐1001 H3 (300 μg·day^−1^ per mouse) started. The control group received the same volume of the diluting medium. Five days after the end of treatment, the lungs were collected and metastatic nodules were counted.

### Statistical analysis

The data were expressed as the means ± SD. Significant differences were assessed using Student's *t*‐test. *P*‐values < 0.05 were considered significant.

## Results

### Antimetastatic activity of AC‐1001 H3

**Figure 1:**
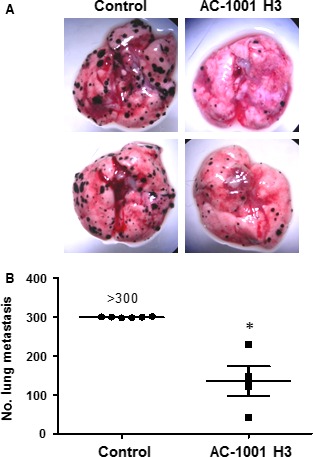
Antimetastatic effects of peptide AC‐1001 H3. Syngeneic B16F10‐Nex2 cells (5 × 10^5^) were injected in C57Bl6 mice (*n* = 6). AC‐1001 H3 peptide was administered intraperitoneally for 10 consecutive days (300 μg·day^−1^ per mouse). Representative images of lungs (A), and the number of nodules in the lungs of control and treated animals (B), are shown. A significant reduction in the number of metastatic nodules was observed (*P* = 0.0006).

We demonstrated in the present work that AC‐1001 H3 has antimetastatic effects using a syngeneic model with B16F10‐Nex2 cells injected intravenously in male C57BL/6 mice. The CDR peptide was administered intraperitoneally for 10 consecutive days at 300 μg·day^−1^ per mouse. After the end of treatment, the lungs were collected (Fig. 1A) and melanotic metastatic nodules were counted. There was a significant difference in the number of nodules between the untreated (control), and peptide‐treated groups (Fig. 1B). Furthermore, the AC‐1001 H3 at a high dose, 25 mg·kg^−1^, was injected for 3 consecutive days on healthy mice, their organs (liver, heart, kidneys, spleen, lung and intestine) were collected and examined for histopathological signs. No alterations were found (data not shown), suggesting the lack of toxicity of AC‐1001 H3, a promising antitumor drug.

### *In vitro* cytotoxicity

**Figure 2:**
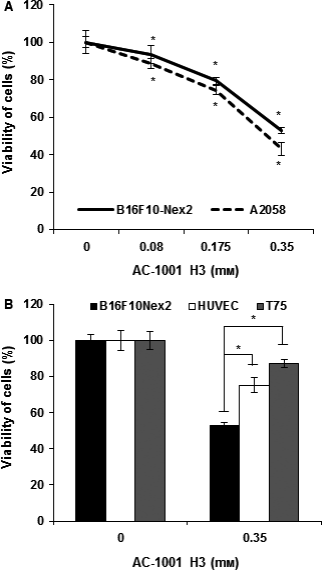
*In vitro* cytotoxic activity of AC‐1001 H3. (A) B16F10‐Nex2 and A2058 cells lines were treated for 18 h with CDR AC‐1001 H3, at concentrations within the solubility limits of the peptide. (B) AC1001 H3 cytotoxicity in nontumorous cell lines in comparison with B16F10‐Nex2 cell line. Cell viability was determined by MTT assay. *P < 0.05.

The *in vitro* cytotoxicity of AC‐1001 H3 was tested on B16F10‐Nex2 cells starting with 10^4^ tumor cells incubated overnight with different concentrations of the peptide. The cell viability was tested using the MTT method. The AC‐1001 H3 decreased cell viability in a dose‐dependent manner and at the highest concentration allowed by its solubility (0.35 mm), the peptide killed 50% of the tumor cells. Similarly to the murine melanoma cells, human A2058 melanoma cells were equally sensitive to the peptide at IC50 (Fig. 2A). A scrambled peptide did not show any cytotoxic activity at the same concentration (data not shown). HUVEC and T75 human endothelial and fibroblast cell lines, respectively, were significantly less affected by the peptide (Fig. 2B). Cultured cells, however, are a controversial control of peptide toxicity, which was not observed *in vivo* at high concentration of the peptide.

We investigated, therefore, the peptide's *in vitro* mechanism of action using the murine melanoma cell line. Since 0.35 mm represents the peptide's solubility limit and also corresponds to its IC50 in B16F10‐Nex2 cells, this concentration was mostly used throughout this work.

### Tumor cell apoptosis

**Figure 3:**
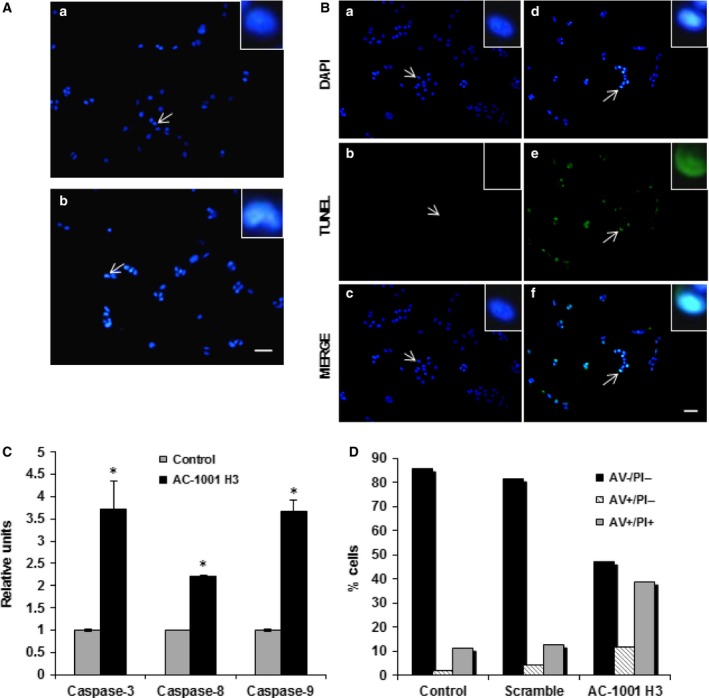
AC‐1001 H3 induces apoptosis. B16F10‐Nex2 cells were treated with AC‐1001 H3 (0.35 mm) and examined for apoptosis hallmarks. (A) Untreated (a) and treated (b) cells were stained with Hoechst 33342. Chromatin condensation was observed in treated cells (arrows indicate cells highlighted in inserts, zoom 700%). Bar, 100 μm. (B) TUNEL assay showing DNA degradation in treated cells (d–f) as compared to untreated cells (a–c); arrows indicate cells highlighted in inserts, zoom (700%). Bar, 100 μm. (C) Caspase‐3, ‐8 and ‐9 activation after peptide treatment, *P < 0.05. (D) Analysis by FACs of peptide‐treated cells labeled with annexin V‐FITC (AV) and propidium iodide (PI).

Using Hoechst 33342, a fluorescent dye that intercalates within DNA, we observed that ~ 60% of peptide‐treated cells had condensed chromatin (Fig. 3A). TUNEL assay, used to analyze DNA fragmentation based on the binding of labeled nucleotides to free 3′‐OH DNA ends generated during apoptosis, undoubtedly revealed that AC‐1001 H3 treatment leads to DNA fragmentation, as seen by the green fluorescence (Fig. 3B).

A colorimetric method was used to measure caspase activity, which has a critical role in the apoptotic pathway 19. Notably, there was a significant increase in caspases 3, 8, and 9 after AC‐1001 H3 treatment, with levels 3.5‐fold, 2‐fold, and 3.5‐fold higher than the untreated controls, respectively (Fig. 3C).

Translocation of phosphatidylserine, a well‐known feature of early apoptotic process, and propidium iodide binding to DNA, a late necrotic event following disintegration of the plasma membrane, were evaluated by flow cytometry with Annexin V and propidium iodide labeling. An increase in annexin V positive cells after AC‐1001 H3 treatment (11.7%) as compared to the untreated control (2.25%) or the scrambled peptide (4.48%), was observed. Double labeled, annexin V, and PI positive cells, however, increased up to 38.9% in treated cells as compared to 11.6% (control) and 13% (scrambled peptide) (Fig. 3D).

**Figure 4:**
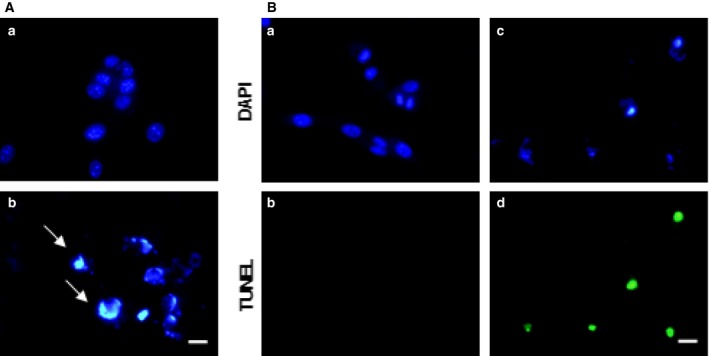
AC‐1001 H3 also induces apoptosis in A2058 cells. Cells were treated with AC‐1001 H3 (0.35 mm) and examined for apoptosis hallmarks. (A) Untreated (a) and treated (b) cells were stained with Hoechst 33342. Chromatin condensation was observed in treated cells (white arrows) Bar, 20 μm. (B) TUNEL assay showing DNA degradation in treated cells (c, d) as compared to untreated cells (a, b). Bar, 20 μm.

AC‐1001 H3 also induced apoptosis in A2058 cells. Cells treated with AC‐1001 H3 (0.35 mm) showed chromatin condensation and positive TUNEL assay reactivity (Fig. 4).

### Superoxide anions formation and mitochondria interference

**Figure 5:**
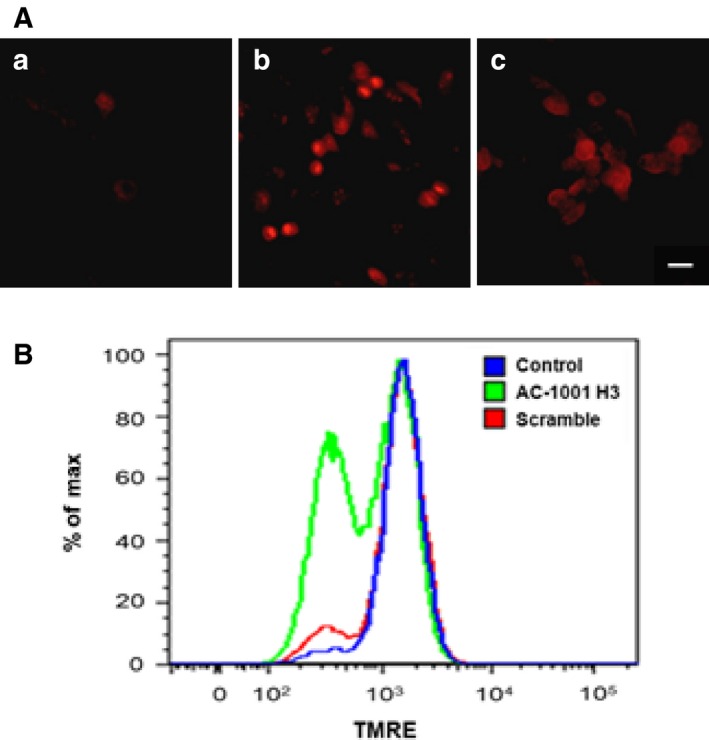
Anion superoxide production and mitochondrial effects induced by AC‐1001 H3. B16F10‐Nex2 cells were treated with AC‐1001 H3 (0.35 mm) for 12–18 h. (A) Anion superoxide production induced by peptide treatment; (a), untreated cells; (b) positive control with 5 mm H_2_O_2_; (c) AC‐1001 H3. Bar, 50 μm. (B) Mitochondrial transmembrane‐potential (Δψm) dissipation. Untreated cells (blue), AC‐1001 H3 (green) and scramble peptide (red).

Reactive oxygen species production was detected by DHE (dihydroethidium), which is oxidized to ethidium by superoxide anions, emitting red fluorescence. Increased superoxide anions formed after AC‐1001 H3 treatment (Fig. 5A).

Dissipation of the mitochondrial membrane potential was estimated by TMRE (tetramethylrhodamine ethyl ester) staining. The dye accumulates in active mitochondria but not in depolarized mitochondria. Flow cytometry analysis showed fluorescence reduction in AC‐1001 H3 treated cells as compared to control and scrambled peptide‐treated cells (Fig. 5B) thus indicating lower TMRE accumulation and mitochondrial membrane depolarization induced by AC‐1001 H3.

### Ultrastructural effects of AC‐1001 H3

**Figure 6:**
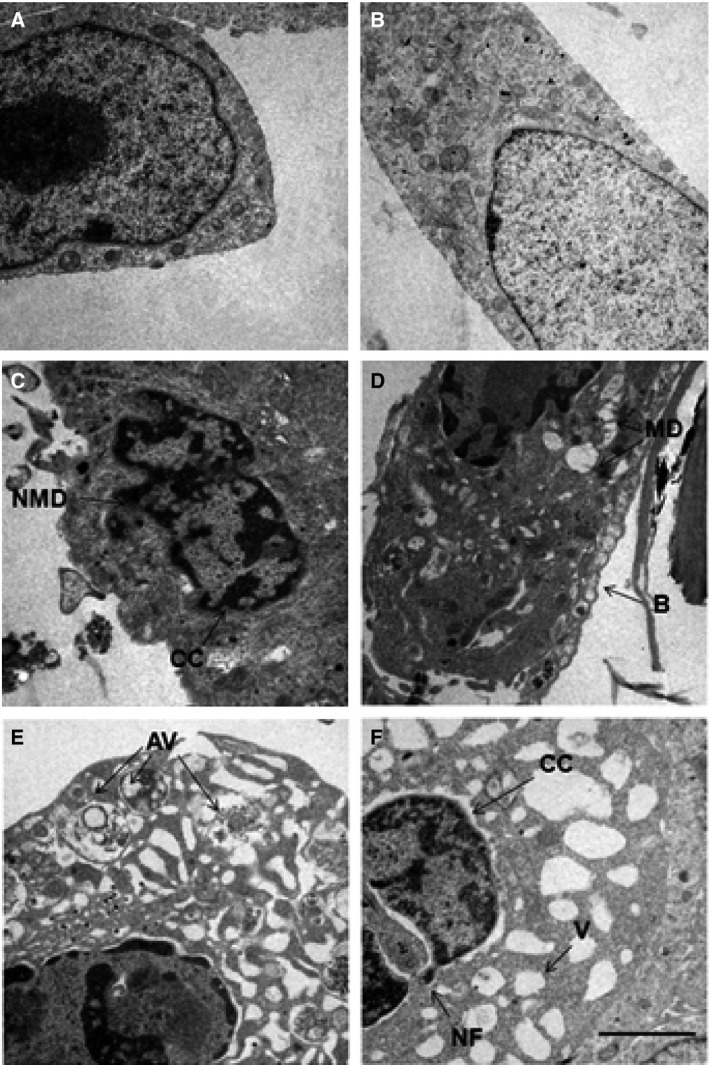
Cell structure effects induced by AC‐1001 H3. B16F10‐Nex2 cells were treated with AC‐1001 H3 (0.35 mm) and observed by transmission electron microscopy. (A, B) untreated cells; (C) nuclear membrane disruption (NMD) and chromatin condensation (CC) in treated cells; (D) mitochondria disintegration (MD) and blebs (B); (E) autophagic vacuoles (AV); (F) nuclear fragmentation (NF), chromatin condensation (CC) and vacuoles (V). Bar, 0.5 μm.

Control and peptide‐treated cells were also analyzed through transmission electron microscopy (TEM; Fig. 6). Chromatin condensation (CC), nuclear fragmentation (NF), nuclear membrane disintegration (MD), formation of cytoplasmic vacuoles (V), and blebs (B) are typical apoptotic morphological alterations that were found and they corroborate biochemical determinations. Further observation, however, showed that in addition to the cytoplasmic vacuolation, structures similar to autophagic vacuoles were also seen.

### Peptide‐induced signs of autophagy

**Figure 7:**
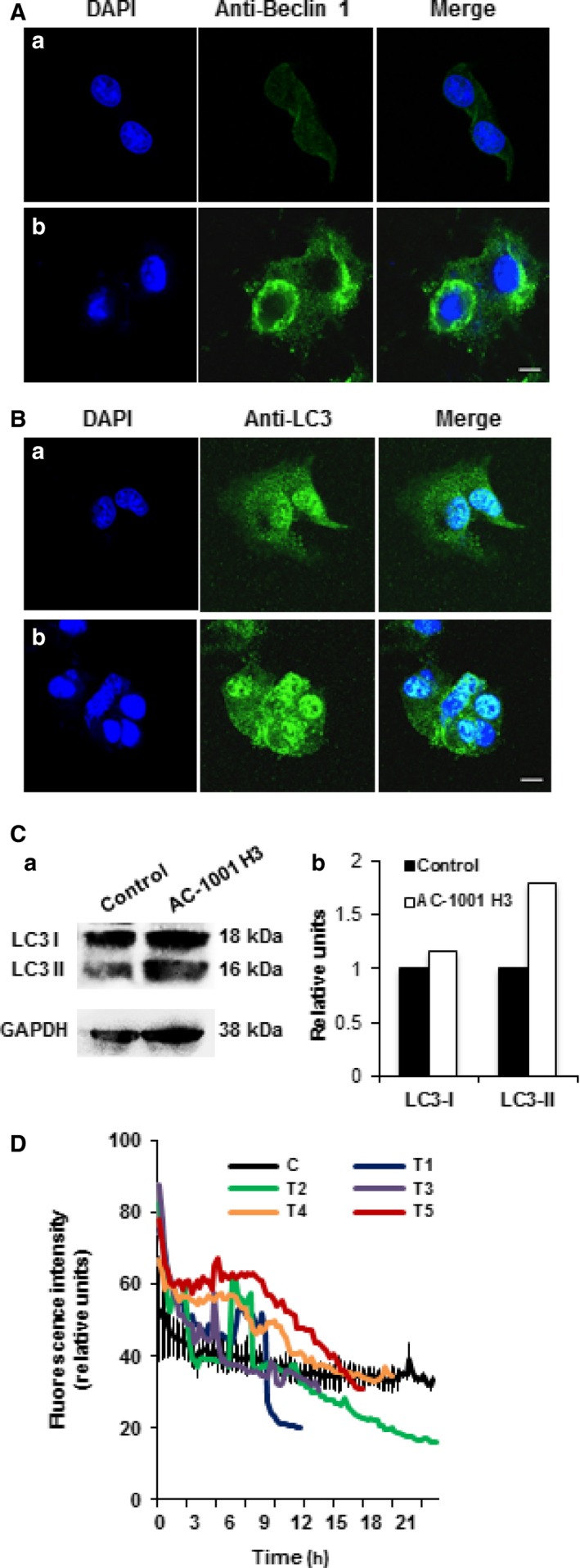
AC‐1001 H3 induces autophagy. B16F10‐Nex2 cells were treated with AC‐1001 H3 (0.35 mm) overnight. Beclin 1 (A) and LC3 (B) proteins were analyzed by confocal microscopy using specific antibodies. (a) untreated and (b) treated cells. Bar, 20 μm. (C) Immunoblotting analysis of LC3‐I (18 kDa) and LC3‐II (16 kDa) expression, using anti‐GAPDH as protein loading control (a). Quantification of band area intensity (b). (D) Cells were incubated with AC‐1001 H3 peptide or untreated control (C) cells and simultaneously labeled with 5 μm LysoTracker Red. Images were taken every 5 min in a confocal microscopy and the fluorescence was measured using imaris software. C (black) – untreated cells; T1–T5 (color) – individually represented cells.

Cells were incubated with antibodies against LC3 and Beclin 1 proteins, which are essential for autophagosome formation. Using confocal microscopy, we demonstrated an enhanced expression of LC3 and Beclin 1 proteins on peptide‐treated cells (Fig. 7A,B).

Since LC3‐II, formed by conjugation with phosphatidylethanolamine, is a marker of the elongation phase of the isolation membrane to capture damaged organelles or cytosolic materials, it indicates an increase in the rate of autophagosome formation 20. Western blotting analysis of LC3 expression (Fig. 7C) showed an increase in the expression of LC3‐II (16 kDa) in AC‐1001 H3 treated cells. Quantification showed a value 1.8‐fold higher than in untreated cells.

We also used Lysotracker Red for labeling acidic organelles in live cells. Using imaris software to quantify the number of acidic organelles based on the cell volume, we did not find a significant difference in control or treated cells, which could indicate poor transition into autolysosomes for degradation of captured materials or a blockade of autophagosome maturation. When the fluorescence intensity was measured as indicated in Fig. 7D, a few peptide‐treated cells showed higher acidity as compared to control cells. Further studies are needed, however, to confirm the autophagy flux in peptide‐treated tumor cells.

### Time‐dependent expression of Beclin 1 and caspase 3

**Figure 8:**
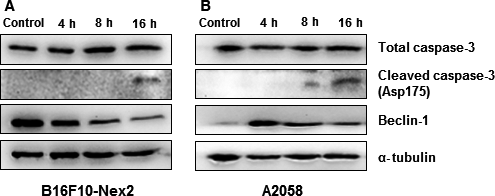
Kinetics of Beclin 1 and caspase 3 expression in melanoma cell lines. B16F10‐Nex2 (A), and A2058 (B) tumor cells (5 × 10^4^) were treated with 0.35 mm of AC1001 peptide for 4, 8 and 16 h. After treatment, proteins in cell lysates were analyzed by western blotting. α‐tubulin was used as loading control. Secondary antibodies were conjugated with IgG horseradish peroxidase.

The expression of Beclin 1 and caspase 3 was followed at 0, 4, 8, and 16 h in both B16F10‐Nex2 and A2058 cell lines incubated with Ac‐1001 H3 (Fig. 8A,B). The endogenous expression of Beclin 1 was high in B16F10‐Nex2 and low in A2058 lysates. In the absence of autophagy Beclin 1 could be bound to Bcl‐2 or Bcl‐XL 21, which are also highly expressed in B16F10 cells 22 (V.S.C. Maia, unpublished result). In A2058 human melanoma cells, the expression of Beclin 1 increased at 4 h incubation with the peptide. In both cases, after 8 h, there was a progressive degradation of Beclin 1 simultaneously with the increasing expression procaspase 3, which cleaves Beclin 1, clearly indicating that the apoptosis process develops after the observed signs of autophagy described above.

### AC‐1001 H3 cell ligand

**Figure 9:**
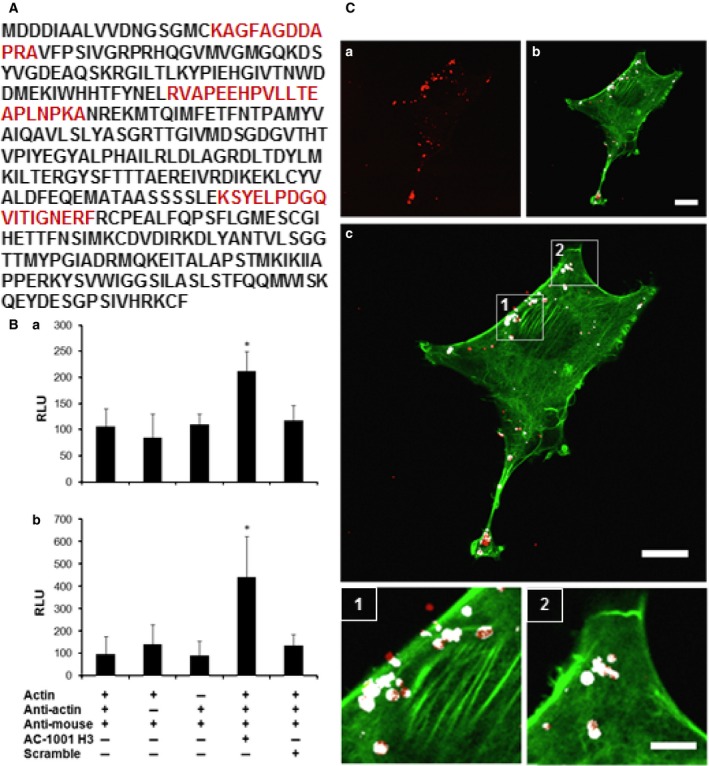
AC‐1001 H3 binding to β‐actin. B16F10‐Nex2 cell lysate was incubated with biotinylated AC‐1001 H3. Proteins were separated in streptavidin‐Agarose and eluted with urea. The eluate was separated in SDS/PAGE, and the single band detected was analyzed by mass spectrometry. (A) Peptides from LC MS/MS leading to β‐actin identification are shown, over the entire sequence, in red. (B) AC‐1001 H3 and β‐actin binding was confirmed by ELISA using G‐actin (a) or F‐actin (b). *P < 0.05. (C) Confocal microscopy showing F‐actin and AC‐1001 H3 co‐localization. B16F10‐Nex2 cells were incubated with peptide‐NPs‐rhodamine (a) according to experimental procedures and also stained with Phalloidin‐FITC. (b) Merge (c) Showing co‐localization points in white and highlighted below (1 and 2). Bar 20 μm (a–c), and 60 μm (1 and 2).

In order to better understand the peptide's mechanism of action, we investigated possible ligands in tumor cells. Lysed B16F10‐Nex2 cells were incubated with biotinylated AC‐1001 H3, passed through a streptavidin‐Agarose column and eluted with 2% urea. The eluate was separated in 10% SDS/PAGE. The only detected band was digested with trypsin and analyzed by Q‐TOF mass spectrometry. We found three peptides that matched with β‐actin, suggesting it to be the main target of AC‐1001 H3 (Fig. 9A). It was confirmed by the strong binding of AC‐1001 H3 peptide to both G‐ and F‐actin in an ELISA assay (Fig. 9B) and by confocal microscopy, using peptide‐NPs‐rhodamine labeling (Fig. 9C). Cells were treated with AC‐1001‐NPs‐Rhodamine (Fig. 9C‐a) solution for 3 h, fixed, permeabilized, and stained with phalloidin‐FITC, used to identify filamentous actin (Fig. 9C‐b). Merged images (Fig. 9C‐c) showed colocalization of AC‐1001 H3 peptide and F‐actin in white.

### Inhibition of tumor cell migration

**Figure 10:**
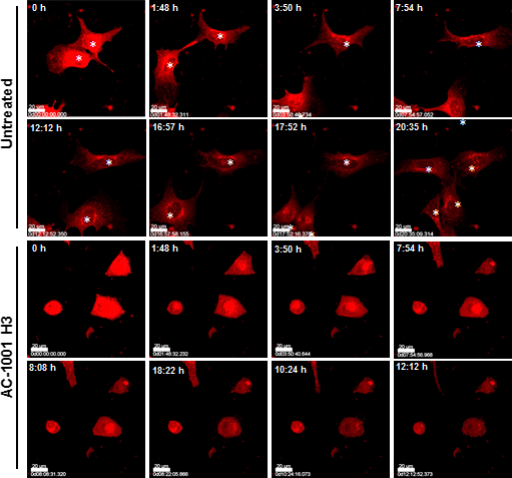
AC‐1001 H3 inhibits cell migration. B16F10‐Nex2 cells treated with 0.35 mm AC‐1001 H3 were labeled with 5 μm lysotracker. Images were taken every 5 min in a confocal microscope in real time. White asterisks indicate untreated cells migrating and duplicating in contrast with treated cells. Bar, 100 μm. The time of incubation in each image is indicated.

Using the images obtained with lysotracker a video was prepared with control and peptide‐treated melanoma cells (Video S1). It was evident that AC‐1001 H3 treatment, by interacting with key components of the cell skeleton, caused cell shrinkage and promptly inhibited cell migration. White asterisks indicate migration and duplication of control cells (Fig. 10), whereas no alterations were seen in peptide‐treated cells.

## Discussion

Peptides have attracted attention as anticancer drugs due to their versatility in terms of synthesis and derivatization. Janin 23 reviewed a range of characterized peptides that have established or potential anticancer use. Mechanisms of action allowed their classification as receptor‐interacting compounds, inhibitors of protein‐protein interaction, enzyme inhibitors, nucleic acid‐interacting compounds. Bhutia and Maiti emphasized the potential of novel peptides, derived from natural sources 9. As reviewed by these authors, anticancer cytotoxicity includes tumor cell necrosis, apoptosis, function blocking, and the inhibition of angiogenesis, besides immune stimulation.

The potential of immunoglobulins as a source of bioactive peptides is based on the pivotal work by Polonelli *et al*. 24 on fragments of an antiidiotypic antibody, in particular an engineered killer peptide that displayed a broad antimicrobial activity 24. This was extended to CDRs from unrelated murine and human monoclonal Abs, which, irrespective of the native Ab specificity, displayed *in vitro*,* in vivo,* and/or *ex vivo* antifungal (*Candida albicans*), antiviral (HIV‐1), and antitumor (melanoma) activities 10, 11. Receptors on target cells for the Ig‐derived peptides and their effects on cell function are slowly being recognized 12, 15, 25, 26. In melanoma cells, CDRs from different monoclonal antibodies bound to protocadherin beta‐13, histone‐1, beta‐actin, tubulin, HSP90, and GPR124 12, 25 (N. Girola and L.R. Travassos, unpublished results). Arruda *et al*. 25 demonstrated that the peptide based on CDR H2 from mAb C7, against *C. albicans*, induced apoptosis in tumor cell lines by β‐actin binding and disturbing actin dynamics. It also displayed an important antimetastatic activity 25.

The potential of the synthetic peptide AC‐1001‐H3 (based on mouse mAb AC‐1001 CDR H3) as an antitumor drug, was explored in this article. Initially we evaluated its activity *in vitro* on tumor and nontumor cell lines, treated overnight at different peptide concentrations. We observed that AC‐1001 H3 was cytotoxic in tumor cells and significantly less effective in nontumor cell lineages.

Using the murine B16F10‐Nex2 melanoma cell line, we focused on the apoptotic death pathway as a possible mechanism for action of the peptide. Apoptosis is a highly coordinated type of cell death triggered by various stimuli, activating either the extrinsic death receptor pathway or the intrinsic mitochondrial pathway. It involves the activation of caspases and a cascade of events that culminate in cell demise 27, 28. Chromatin condensation, DNA fragmentation, activation of caspases‐3, ‐8, and ‐9, external translocation of phosphatidylserine, increased ROS production and dissipation of the mitochondrial transmembrane potential were effects induced by the peptide strongly suggesting the intrinsic apoptosis pathway. TEM, considered as the ‘gold standard’ assay to confirm the cell death mechanism 29, was also performed. Alterations in the cell ultrastructure were observed including condensed chromatin, nuclear membrane disintegration, nuclear fragmentation, and plasma membrane blebbing, some well‐known features of apoptosis 28, 30, 31. The human melanoma cell line A2058 was equally affected by AC 1001 H3 at the same cytotoxic concentration showing condensed chromatin and a positive TUNEL assay for DNA degradation.

Features of different types of cell death can overlap in certain cases, so that different assays are important to allow an accurate interpretation 27. Our results showed both AV+/PI− and AV+/PI+ cells upon AC‐1001 H3 treatment, suggesting necrosis, with plasmatic membrane disintegration. In fact, cultured cells undergoing apoptosis *in vitro* will eventually undergo secondary time‐dependent necrosis 27. The AV+/PI+ cells, as observed, may represent a late apoptosis, with alterations in the membrane's integrity 32 or an aponecrosis, combining apoptosis, and necrosis 33. Since membrane alterations are relatively common in adherent cells cultured *in vitro*, annexin‐V and PI positive cells in a flow cytometry analysis are usually regarded as apoptotic 34, 35.

As to caspase activation, the peptide induced the upstream initiators caspase‐8 and ‐9 and the downstream execution caspase‐3, with a more emphatic increase in caspase‐9 and ‐3. Caspase‐8 is associated with the extrinsic pathway of apoptosis, while caspase‐9 is related to the intrinsic pathway 19. As previously described, there may be a crosstalk between both apoptotic pathways mediated by Bid 36, 37. Alternatively, caspase‐8 could be triggered by ROS, mediating the mitochondrial pathway and bypassing CD95/Fas engagement 25, 38, 39. It has also been reported the possibility of an interdependence of caspase‐2, ‐8, ‐9, and ‐3 in an apoptotic process mediated by ROS 17, 40. Once we also had mitochondrial transmembrane‐potential dissipation and increased ROS production, the apoptosis induced by AC‐1001 H3 likely occurred via the intrinsic pathway and caspase‐8 activation was induced by ROS.

Transmission electron microscopy analysis additionally revealed a massive cell vacuolization and organelles within some vesicles, seemingly autophagy suggesting images 41, 42, 43. Through autophagy, proteins and organelles are sequestered into double membrane vesicles (autophagosomes) and are subsequently delivered to the lysosomes for degradation 44. It is an evolutionary conserved process that occurs in all eukaryotic cells and is characterized by the accumulation of autophagic vesicles 45. Studies in yeast allowed the identification of evolutionary conserved autophagy‐related (Atg) genes that control major steps in the process 46. Beclin 1 is a mammalian ortholog of yeast Atg6 and is required for autophagosome formation. Microtubule‐associated protein 1 light chain 3 (LC3) is the ortholog of yeast Atg8 45.

We have shown by fluorescence microscopy the increased expression of Beclin 1 and LC3 expression after peptide treatment. Interestingly, some anticancer drugs that induce autophagy may act through the up‐regulation of Beclin 1, as exemplified for the ceramide‐mediated process induced by tamoxifen 47. Kabeya *et al*. described that LC3‐I and LC3‐II are generated by post‐translational modifications and that LC3‐I (cytosolic) is converted to LC3‐II (membrane bound), localized in autophagosome membranes. There is a correlation between LC3‐II and elongation of the isolation membrane, hence of autophagosome formation, which makes LC3‐II a good marker for autophagosomes 20. To support the previous result, we also showed by western blotting, two bands at 18 kDa (LC3‐I) and 16 kDa (LC3‐II). Their quantification showed a substantial increase in LC3‐II expression, which may indicate enhanced autophagosome assembly. Since the autophagic flux was not further explored it is unclear whether, in this experiment, increased levels of LC3‐II might otherwise reflect accumulation of autophagosomes due to downstream blockade of autolysosome formation 48. Such interference in the maturation step could well occur by the mechanism of action of AC 1001 H3, primarily based on β‐actin binding. In fact, both G‐ and F‐actin are ligands to the peptide. By targeting actin, apart from the effects on microtubule distribution 49, the peptide might also target actin‐related proteins of the dynactin complex 50 which, with dynein, drive autophagosomes on microtubules to the cell center where most lysosomes are 51. Such hypotheses require further experiments for validation. Peptide‐treated cells stained with lysotracker red, a lysosomotropic agent, did not show an increase in the number of acidic organelles, but some individual cells had a more intense fluorescence measurement. These results describe autophagy induction signs but are insufficient to confirm a complete autophagy flux in response to the CDR peptide that acts on the actin framework, inhibits cell migration (Video S1) and elicits tumor cell apoptosis. The signs of autophagy clearly precede the apoptosis process as shown by Beclin 1 expression in both B1F10‐Nex2 and A2058 cell lines as well as its degradation simultaneously with caspase 3 expression. Caspase‐mediated cleavage of beclin 1 promotes the crosstalk between apoptosis and autophagy 21.

One of the major functions of autophagy is to keep cells alive under stressful ‘life‐threatening conditions’ as stated by Kroemer and Levine 52. A significant decrease in tumor growth in mice treated with the autophagy inhibitor hydroxychloroquine (HCQ) was reported 53. These observations agree with other reports showing that HCQ inhibits *in vitro* cell growth and *in vivo* tumor growth via induction of apoptosis. Inhibition of autophagy promotes cancer cell death 54 and potentiates anticancer treatments. Several articles have addressed the combination of apoptosis and autophagy 30, 42, 43, 55, 56, 57. Finally and most importantly, we studied the peptide effect *in vivo* in a syngeneic model. AC‐1001 H3 was able to inhibit metastasis, leading to a significant decrease in the number of lung nodules in mice treated with the peptide in comparison to the untreated control. No toxic effects were observed during the treatment protocol, even by histopathological inspection of mice organs. Moreover, injection of a high dose of the peptide into healthy animals also did not show histological alterations.

In conclusion, we suggest that AC‐1001 H3 targets β‐actin in melanoma cells, induces apoptosis after signs of autophagy in tumor cells while exerting little cytotoxicity in non‐tumorigenic cells *in vitro* and no observed toxicity *in vivo* in healthy animals. It exhibits a potent antimetastatic effect *in vivo* and can be considered a promising candidate for drug development in cancer therapy.

## Author contributions

ANR, DCA, CRF, MHM, VM, CFF, DBT, PISJ, acquired and analyzed data. FR and RAM performed experiments using confocal microscopy and NG did the western blotting experiments. LP, LRT, and DCA conceived and supervised the project. ANR, DCA, LP, and LRT wrote and revised the manuscript.

## Supporting information


**Video S1.** AC‐1001 H3 causes cell shrinkage and inhibits cell migration. B16F10‐Nex2 cells were treated with 0.35 mm AC‐1001 H3, simultaneously labeled with 5 μm lysotracker and examined in a confocal microscopy. Images were taken during 22 h every 5 min and a video setting was prepared using imaris software.

## References

[1] Bandarchi B , Jabbari CA , Vedadi A and Navab R (2013) Molecular biology of normal melanocytes and melanoma cells. J Clin Pathol 66, 644–648. 23526597 10.1136/jclinpath-2013-201471

[2] Silva JH , Sá BC , Avila AL , Landman G and Duprat Neto JP (2011) Atypical mole syndrome and dysplastic nevi: identification of populations at risk for developing melanoma – review article. Clinics (Sao Paulo) 66, 493–499. 21552679 10.1590/S1807-59322011000300023 PMC3072014

[3] Kuphal S and Bosserhoff A (2009) Recent progress in understanding the pathology of malignant melanoma. J Pathol 219, 400–409. 19771562 10.1002/path.2617

[4] Gray‐Schopfer V , Wellbrock C and Marais R (2007) Melanoma biology and new targeted therapy. Nature 445, 851–857. 17314971 10.1038/nature05661

[5] Miller AJ and Mihm MC Jr (2006) Melanoma. N Engl J Med 355, 51–65. 16822996 10.1056/NEJMra052166

[6] Boiko AD , Razorenova OV , van de Rijn M , Swetter SM , Johnson DL , Ly DP , Butler PD , Yang GP , Joshua B , Kaplan MJ *et al* (2010) Human melanoma‐initiating cells express neural crest nerve growth factor receptor CD271. Nature 466, 133–137. 20596026 10.1038/nature09161 PMC2898751

[7] Scheier B , Amaria R , Lewis K and Gonzalez R (2011) Novel therapies in melanoma. Immunotherapy 3, 1461–1469. 22091682 10.2217/imt.11.136

[8] Kyi C and Postow MA (2014) Checkpoint blocking antibodies in cancer immunotherapy. FEBS Lett 588, 368–376. 24161671 10.1016/j.febslet.2013.10.015

[9] Bhutia SK and Maiti TK (2008) Targeting tumors with peptides from natural sources. Trends Biotechnol 26, 210–217. 18295917 10.1016/j.tibtech.2008.01.002

[10] Polonelli L , Pontón J , Elguezabal N , Moragues MD , Casoli C , Pilotti E , Ronzi P , Dobroff AS , Rodrigues EG , Juliano MA *et al* (2008) Antibody complementarity‐determining regions (CDRs) can display differential antimicrobial, antiviral and antitumor activities. PLoS One 3, e2371. 18545659 10.1371/journal.pone.0002371 PMC2396520

[11] Magliani W , Conti S , Cunha RL , Travassos LR and Polonelli L (2009) Antibodies as crypts of antiinfective and antitumor peptides. Curr Med Chem 16, 2305–2323. 19519392 10.2174/092986709788453104

[12] Dobroff AS , Rodrigues EG , Juliano MA , Friaça DM , Nakayasu ES , Almeida IC , Mortara RA , Jacysyn JF , Amarante‐Mendes GP , Magliani W *et al* (2010) Differential antitumor effects of IgG and IgM monoclonal antibodies and their synthetic complementarity‐determining regions directed to new targets of B16F10‐Nex2 melanoma cells. Transl Oncol 3, 204–217. 20689762 10.1593/tlo.09316 PMC2915412

[13] Heap CJ , Wang Y , Pinheiro TJT , Reading SA , Jennings KR and Dimmock NJ (2005) Analysis of a 17‐amino acid residue, virus‐neutralizing microantibody. J Gen Virol 86, 1791–1800. 15914858 10.1099/vir.0.80812-0

[14] Nickerson KG , Tao MH , Chen HT , Larrick J and Kabat EA (1995) Human and mouse monoclonal antibodies to blood group A substance, which are nearly identical immunochemically, use radically different primary sequences. J Biol Chem 270, 12457–12465. 7759488 10.1074/jbc.270.21.12457

[15] Gabrielli E , Pericolini E , Cenci E , Ortelli F , Magliani W , Ciociola T , Bistoni F , Conti S , Vecchiarelli A and Polonelli L (2009) Antibody complementarity‐determining regions (CDRs): a bridge between adaptive and innate immunity. PLoS One 4, e8187. 19997599 10.1371/journal.pone.0008187 PMC2781551

[16] Dobroff AS , Rodrigues EG , Moraes JZ and Travassos LR (2002) Protective, anti‐tumor monoclonal antibody recognizes a conformational epitope similar to melibiose at the surface of invasive murine melanoma cells. Hybrid Hybridomics 21, 321–331. 12470474 10.1089/153685902761022661

[17] Massaoka MH , Matsuo AL , Figueiredo CR , Farias CF , Girola N , Arruda DC , Scutti JA , Romoff P , Favero OA , Ferreira MJ *et al* (2012) Jacaranone induces apoptosis in melanoma cells via ROS‐mediated downregulation of Akt and p38 MAPK activation and displays antitumor activity *in vivo* . PLoS One 7, e38698. 22701695 10.1371/journal.pone.0038698 PMC3368838

[18] Shevchenko A , Wilm M , Vorm O and Mann M (1996) Mass spectrometric sequencing of proteins silver‐stained polyacrylamide gels. Anal Chem 68, 850–858. 8779443 10.1021/ac950914h

[19] Fiandalo MV and Kyprianou N (2012) Caspase control: protagonists of cancer cell apoptosis. Exp Oncol 34, 165–175. 23070001 PMC3721730

[20] Kabeya Y , Mizushima N , Ueno T , Yamamoto A , Kirisako T , Noda T , Kominami E , Ohsumi Y and Yoshimori T (2000) LC3, a mammalian homologue of yeast Apg8p, is localized in autophagosome membranes after processing. EMBO J 19, 5720–5728. 11060023 10.1093/emboj/19.21.5720 PMC305793

[21] Kang R , Zeh HJ , Lotze MT and Tang D (2011) The beclin‐1 network regulates autophagy and apoptosis. Cell Death Differ 18, 571–580. 21311563 10.1038/cdd.2010.191 PMC3131912

[22] Faião‐Flores F , Coelho PRP , Toledo Arruda‐Neto JD , Maria‐Engler SS , Tiago M , Capelozzi VL , Giorgi RR and Maria DA (2013) Apoptosis through Bcl‐2/Bax and cleaved caspase up‐regulation in melanoma treated by boron neutron capture therapy. PLoS One 8, e59639. 23527236 10.1371/journal.pone.0059639 PMC3603877

[23] Janin YL (2003) Peptides with anticancer use or potential. Amino Acids 25, 1–40. 12836056 10.1007/s00726-002-0349-x

[24] Polonelli L , Magliani W , Conti S , Bracci L , Lozzi L , Neri P , Adriani D , De Bernardis F and Cassone A (2003) Therapeutic activity of an engineered synthetic killer antiidiotypic antibody fragment against experimental mucosal and systemic candidiasis. Infect Immun 71, 6205–6212. 14573638 10.1128/IAI.71.11.6205-6212.2003 PMC219587

[25] Arruda DC , Santos LC , Melo FM , Pereira FV , Figueiredo CR , Matsuo AL , Mortara RA , Juliano MA , Rodrigues EG , Dobroff AS *et al* (2012) β‐actin‐binding complementarity‐determining region 2 of variable heavy chain from monoclonal antibody C7 induces apoptosis in several human tumor cells and is protective against metastatic melanoma. J Biol Chem 287, 14912–14922. 22334655 10.1074/jbc.M111.322362 PMC3340220

[26] Cenci E , Pericolini E , Mencacci A , Conti S , Magliani W , Bistoni F , Polonelli L and Vecchiarelli A (2006) Modulation of phenotype and function of dendritic cells by a therapeutic synthetic killer peptide. J Leukoc Biol 79, 40–45. 16244115 10.1189/jlb.0205113

[27] Elmore S (2007) Apoptosis: a review of programmed cell death. Toxicol Pathol 35, 495–516. 17562483 10.1080/01926230701320337 PMC2117903

[28] Thornberry NA and Lazebnik Y (1998) Caspases: enemies within. Science 281, 1312–1316. 9721091 10.1126/science.281.5381.1312

[29] White MK and Cinti C (2004) A morphologic approach to detect apoptosis based on electron microscopy. Methods Mol Biol 285, 105–111. 15269403 10.1385/1-59259-822-6:105

[30] Visagie MH and Joubert AM (2011) 2‐Methoxyestradiol‐bis‐sulfamate induces apoptosis and autophagy in a tumorigenic breast epithelial cell line. Mol Cell Biochem 357, 343–352. 21656128 10.1007/s11010-011-0905-3

[31] Croft DR , Coleman ML , Li S , Robertson D , Sullivan T , Stewart CL and Olson MF (2005) Actin‐myosin‐based contraction is responsible for apoptotic nuclear disintegration. J Cell Biol 168, 245–255. 15657395 10.1083/jcb.200409049 PMC2171584

[32] Hui X , Chen H , Zhang S , Ma X , Wang X and Huang B (2011) Antitumor activities of recombinant human interferon (IFN)‐λ1 *in vitro* and in xenograft models *in vivo* for colon cancer. Cancer Lett 311, 141–151. 21872388 10.1016/j.canlet.2011.07.004

[33] Formigli L , Papucci L , Tani A , Schiavone N , Tempestini A , Orlandini GE , Capaccioli S and Orlandini SZ (2000) Aponecrosis: morphological and biochemical exploration of a syncretic process of cell death sharing apoptosis and necrosis. J Cell Physiol 182, 41–49. 10567915 10.1002/(SICI)1097-4652(200001)182:1<41::AID-JCP5>3.0.CO;2-7

[34] Han YX and Liang DY (2012) The role of the tumor suppressor RUNX3 in giant cell tumor of the bone. Int J Oncol 40, 673–678. 22076387 10.3892/ijo.2011.1249

[35] Morello S , Sorrentino R , Montinaro A , Luciano A , Maiolino P , Ngkelo A , Arra C , Adcock IM and Pinto A (2011) NK1.1 cells and CD8 T cells mediate the antitumor activity of Cl‐IB‐MECA in a mouse melanoma model. Neoplasia 13, 365–373. 21472141 10.1593/neo.101628 PMC3071085

[36] Winter E , Chiaradia LD , Silva AH , Nunes RJ , Yunes RA and Creczynski‐Pasa TB (2014) Involvement of extrinsic and intrinsic apoptotic pathways together with endoplasmic reticulum stress in cell death induced by naphthylchalcones in a leukemic cell line: advantages of multi‐target action. Toxicol In Vitro 28, 769–777. 24583196 10.1016/j.tiv.2014.02.002

[37] Yin XM (2000) Signal transduction mediated by Bid, a pro‐death Bcl‐2 family proteins, connects the death receptor and mitochondria apoptosis pathways. Cell Res 10, 161–167. 11032168 10.1038/sj.cr.7290045

[38] Kim BM , Choi YJ , Han Y , Yun YS and Hong SH (2009) N,N‐dimethyl phytosphingosine induces caspase‐8‐dependent cytochrome c release and apoptosis through ROS generation in human leukemia cells. Toxicol Appl Pharmacol 239, 87–97. 19481559 10.1016/j.taap.2009.05.020

[39] Giammarioli AM , Maselli A , Casagrande A , Gambardella L , Gallina A , Spada M , Giovannetti A , Proietti E , Malorni W and Pierdominici M (2008) Pyrimethamine induces apoptosis of melanoma cells via a caspase and cathepsin double‐edged mechanism. Cancer Res 68, 5291–5300. 18593930 10.1158/0008-5472.CAN-08-0222

[40] Prasad V , Chandele A , Jagtap JC , Sudheer Kumar P and Shastry P (2006) ROS‐triggered caspase 2 activation and feedback amplification loop in beta‐carotene‐induced apoptosis. Free Radic Biol Med 41, 431–442. 16843824 10.1016/j.freeradbiomed.2006.03.009

[41] van den Boorn JG , Picavet DI , van Swieten PF , van Veen HA , Konijnenberg D , van Veelen PA , van Capel T , Jong EC , Reits EA , Drijfhout JW *et al* (2011) Skin‐depigmenting agent monobenzone induces potent T‐cell autoimmunity toward pigmented cells by tyrosinase haptenation and melanosome autophagy. J Invest Dermatol 131, 1240–1251. 21326294 10.1038/jid.2011.16

[42] Francisco R , Pérez‐Perarnau A , Cortés C , Gil J , Tauler A and Ambrosio S (2012) Histone deacetylase inhibition induces apoptosis and autophagy in human neuroblastoma cells. Cancer Lett 318, 42–52. 22186300 10.1016/j.canlet.2011.11.036

[43] Cirstea D , Hideshima T , Rodig S , Santo L , Pozzi S , Vallet S , Ikeda H , Perrone G , Gorgun G , Patel K *et al* (2010) Dual inhibition of akt/mammalian target of rapamycin pathway by nanoparticle albumin‐bound‐rapamycin and perifosine induces antitumor activity in multiple myeloma. Mol Cancer Ther 9, 963–975. 20371718 10.1158/1535-7163.MCT-09-0763 PMC3096071

[44] Behrends C , Sowa ME , Gygi SP and Harper JW (2010) Network organization of the human autophagy system. Nature 466, 68–76. 20562859 10.1038/nature09204 PMC2901998

[45] Shintani T and Klionsky DJ (2004) Autophagy in health and disease: a double‐edged sword. Science 306, 990–995. 15528435 10.1126/science.1099993 PMC1705980

[46] Levine B and Klionsky DJ (2004) Development by self‐digestion: molecular mechanisms and biological functions of autophagy. Dev Cell 6, 463–477. 15068787 10.1016/s1534-5807(04)00099-1

[47] Scarlatti F , Bauvy C , Ventruti A , Sala G , Cluzeaud F , Vandewalle A , Ghidoni R and Codogno P (2004) Ceramide‐mediated macroautophagy involves inhibition of protein kinase B and up‐regulation of beclin 1. J Biol Chem 279, 18384–18391. 14970205 10.1074/jbc.M313561200

[48] Mizushima N , Yoshimori T and Levine B (2010) Methods in mammalian autophagy research. Cell 140, 313–326. 20144757 10.1016/j.cell.2010.01.028 PMC2852113

[49] Rodriguez OC , Schaefer AW , Mandato CA , Forster P , Bement WM and Waterman‐Storer CM (2003) Conserved microtubule‐actin interactions in cell movement and morphogenesis. Nat Cell Biol 5, 599–609. 12833063 10.1038/ncb0703-599

[50] Schroer TA (1994) New insights into the interaction of cytoplasmic dynein with the actin‐related protein, Arp1. J Cell Biol 127, 1–4. 7929555 10.1083/jcb.127.1.1 PMC2120195

[51] Kimura S , Noda T and Yoshimori T (2008) Dynein‐dependent movement of autophagosomes mediates efficient encounters with lysosomes. Cell Struct Funct 33, 109–122. 18388399 10.1247/csf.08005

[52] Kroemer G and Levine B (2008) Autophagic cell death: the story of a misnomer. Nat Rev Mol Cell Biol 9, 1004–1010. 18971948 10.1038/nrm2527 PMC2727358

[53] Noman MZ , Janji B , Kaminska B , Moer KV , Pierson S , Przanowski P , Buart S , Berchem G , Romero P , Mami‐Chouaib F *et al* (2011) Blocking hypoxia‐induced autophagy in tumors restores cytotoxic T‐cell activity and promotes regression. Cancer Res 71, 5976–5986. 21810913 10.1158/0008-5472.CAN-11-1094

[54] Rubinsztein DC , Gestwicki JE , Murphy LO and Klionsky DJ (2007) Potential therapeutic applications of autophagy. Nat Rev Drug Discov 6, 304–312. 17396135 10.1038/nrd2272

[55] Ait‐Mohamed O , Battisti V , Joliot V , Fritsch L , Pontis J , Medjkane S , Redeuilh C , Lamouri A , Fahy C , Rholam M *et al* (2011) Acetonic extract of *Buxus sempervirens* induces cell cycle arrest, apoptosis and autophagy in breast cancer cells. PLoS One 6, e24537. 21935420 10.1371/journal.pone.0024537 PMC3174189

[56] Liu KS , Liu H , Qi JH , Liu QY , Liu Z , Xia M , Xing GW , Wang SX and Wang YF (2012) SNX‐2112, an Hsp90 inhibitor, induces apoptosis and autophagy via degradation of Hsp90 client proteins in human melanoma A‐375 cells. Cancer Lett 318, 180–188. 22182451 10.1016/j.canlet.2011.12.015

[57] González‐Polo RA , Boya P , Pauleau AL , Jalil A , Larochette N , Souquère S , Eskelinen EL , Pierron G , Saftig P and Kroemer G (2005) The apoptosis/autophagy paradox: autophagic vacuolization before apoptotic death. J Cell Sci 118 (Pt 14), 3091–3102. 15985464 10.1242/jcs.02447

